# The Pseudophosphatase MK-STYX Physically and Genetically Interacts with the Mitochondrial Phosphatase PTPMT1

**DOI:** 10.1371/journal.pone.0093896

**Published:** 2014-04-07

**Authors:** Natalie M. Niemi, Juliana L. Sacoman, Laura M. Westrate, L. Alex Gaither, Nathan J. Lanning, Katie R. Martin, Jeffrey P. MacKeigan

**Affiliations:** 1 Laboratory of Systems Biology, Van Andel Research Institute, Grand Rapids, Michigan, United States of America; 2 Developmental and Molecular Pathways Department, Novartis Institutes for BioMedical Research, Cambridge, Massachusetts, United States of America; University of Iowa, United States of America

## Abstract

We previously performed an RNA interference (RNAi) screen and found that the knockdown of the catalytically inactive phosphatase, MK-STYX [MAPK (mitogen-activated protein kinase) phospho-serine/threonine/tyrosine-binding protein], resulted in potent chemoresistance. Our follow-up studies demonstrated that knockdown of MK-STYX prevents cells from undergoing apoptosis through a block in cytochrome c release, but that MK-STYX does not localize proximal to the molecular machinery currently known to control this process. In an effort to define its molecular mechanism, we utilized an unbiased proteomics approach to identify proteins that interact with MK-STYX. We identified the mitochondrial phosphatase, PTPMT1 (PTP localized to mitochondrion 1), as the most significant and unique interaction partner of MK-STYX. We previously reported that knockdown of PTPMT1, an important component of the cardiolipin biosynthetic pathway, is sufficient to induce apoptosis and increase chemosensitivity. Accordingly, we hypothesized that MK-STYX and PTPMT1 interact and serve opposing functions in mitochondrial-dependent cell death. We confirmed that MK-STYX and PTPMT1 interact in cells and, importantly, found that MK-STYX suppresses PTPMT1 catalytic activity. Furthermore, we found that knockdown of PTPMT1 resensitizes MK-STYX knockdown cells to chemotherapeutics and restores the ability to release cytochrome c. Taken together, our data support a model in which MK-STYX controls apoptosis by negatively regulating PTPMT1. Given the important role of PTPMT1 in the production of cardiolipin and other phospholipids, this raises the possibility that dysregulated mitochondrial lipid metabolism may facilitate chemoresistance.

## Introduction

Chemoresistance in primary and recurrent tumors is a significant challenge commonly encountered in the clinic, often resulting in patient mortality. The majority of currently utilized chemotherapeutic agents function through the induction of an intrinsic cell death program termed apoptosis. Evasion of apoptosis has been recognized as a hallmark of cancer, and as such, is critical to disease manifestation and progression [1]. As chemoresistance may stem from an inability to induce the apoptotic program, identifying novel proteins and pathways involved in this cellular process is paramount to treating recurrent and resistant tumors.

In an attempt to identify novel regulators of the apoptotic pathway, we previously performed an RNAi screen targeting all known and putative kinases and phosphatases in the human genome [2]. Knockdown of MK-STYX (STYXL1), a poorly characterized dual specificity phosphatase (DUSP), resulted in a potent resistance to chemotherapeutic-induced cell death. Interestingly, MK-STYX is predicted to be catalytically inactive due to a naturally occurring substitution at a critical residue within its active site [3]. At the time of our initial screen, little else was known regarding the function of this gene.

In a follow-up study, we demonstrated that small interfering RNA (siRNA)-mediated knockdown of MK-STYX induces robust chemoresistance to multiple cytotoxic death-inducing agents, such as paclitaxel, cisplatin, and etoposide [4]. We found that the loss of MK-STYX blocks cytochrome c release, a critical and rate-limiting step in apoptosis. The release of pro-apoptotic intramitochondrial proteins, including cytochrome c, is mediated by the BCL-2 (B-Cell CLL/Lymphoma 2) family of proteins [5]. Upon activation, effector proteins BAX (BCL2-associated X Protein) or BAK (BCL2-Antagonist/Killer 1) homooligomerize, destabilizing the outer mitochondrial membrane (OMM) and allowing the efflux of pro-apoptotic proteins normally localized within the inner mitochondrial membrane space (IMS) [6]. Based on our robust chemoresistance phenotype in the presence of MK-STYX knockdown, we hypothesized that the loss of this single gene phenocopies the dual loss of BAX/BAK, therefore disrupting mitochondrial outer membrane permeabilization (MOMP) and facilitating chemoresistance.

To determine whether MK-STYX directly affects BAX/BAK oligomerization, we determined its subcellular localization, which we found to be mitochondrial [4]. Interestingly, MK-STYX does not reside at the OMM, but rather is associated with the mitoplast (inner mitochondrial membrane (IMM) and mitochondrial matrix). Thus, MK-STYX loss does not seem to block apoptosis through direct inhibition of the pro-apoptotic BCL-2 proteins, as it is not physically close to the responsible molecular machinery. Instead, MK-STYX appears to regulate mitochondrial susceptibility to apoptotic agents in a fashion distinct from currently characterized mechanisms.

Due to the non-canonical nature of this regulation, we sought to identify interaction partners of MK-STYX to help define its molecular function. To do this, we utilized an unbiased proteomics approach to identify proteins interacting with MK-STYX. This study identified the mitochondrial phosphatase, PTPMT1, as the most significant and unique interaction partner of MK-STYX. PTPMT1 is a catalytically active DUSP located within the IMM, with its phosphatase domain facing the matrix [7]. It is thus associated with the mitoplast compartment of the mitochondria, and based on our previous data, could interact with MK-STYX *in vivo*. Interestingly, other catalytically inactive phosphatases (pseudophosphatases) in the human genome often regulate the catalysis, localization, and/or function of active phosphatases [8], [9], [10], [11], [12]. Therefore, the shared localization of these two proteins (mitoplast associated), as well as a potential pseudophosphatase regulatory mechanism, led us to further explore this interaction.

Here, we demonstrate that MK-STYX and PTPMT1 interact on a genetic, physical, and functional level. We verify the initial proteomics-based identification of the MK-STYX – PTPMT1 interaction using co-immunoprecipitation. Furthermore, we show that MK-STYX directly suppresses the catalytic activity of PTPMT1 using purified proteins *in vitro*. To determine the genetic nature of this interaction, we performed single and combinatorial siRNA transfections of PTPMT1 and MK-STYX. Knockdown of PTPMT1 resensitized MK-STYX-depleted cells to chemotherapeutic treatment and cytochrome c release. Overall, these data suggest a model whereby MK-STYX inhibits PTPMT1 phosphatase activity to modulate cellular viability. Because PTPMT1 is a key regulator of phospholipid biosynthesis, in particular cardiolipin, these data suggest that dysregulated mitochondrial lipid metabolism could be a novel mechanism by which cancer cells can resist therapeutic treatments.

## Results

### A proteomic screen identifies PTPMT1 as a MK-STYX interactor

To more completely understand its cellular and molecular function, we sought to identify novel protein interaction partners of MK-STYX. We utilized an unbiased proteomics approach in which tandem affinity purification (TAP) was followed by mass spectrometry (MS). The TAP-MS approach is appropriate to identify candidate interaction partners, as it has been shown to identify multiprotein complexes under near physiological conditions [13], [14]. MK-STYX was tagged at both the amino (N)- and carboxyl (C)-terminus and expressed in HeLa cells using a retroviral-based system. MK-STYX expression was titrated to near endogenous levels through the adjustment of infection multiplicity.

**Figure 1:**
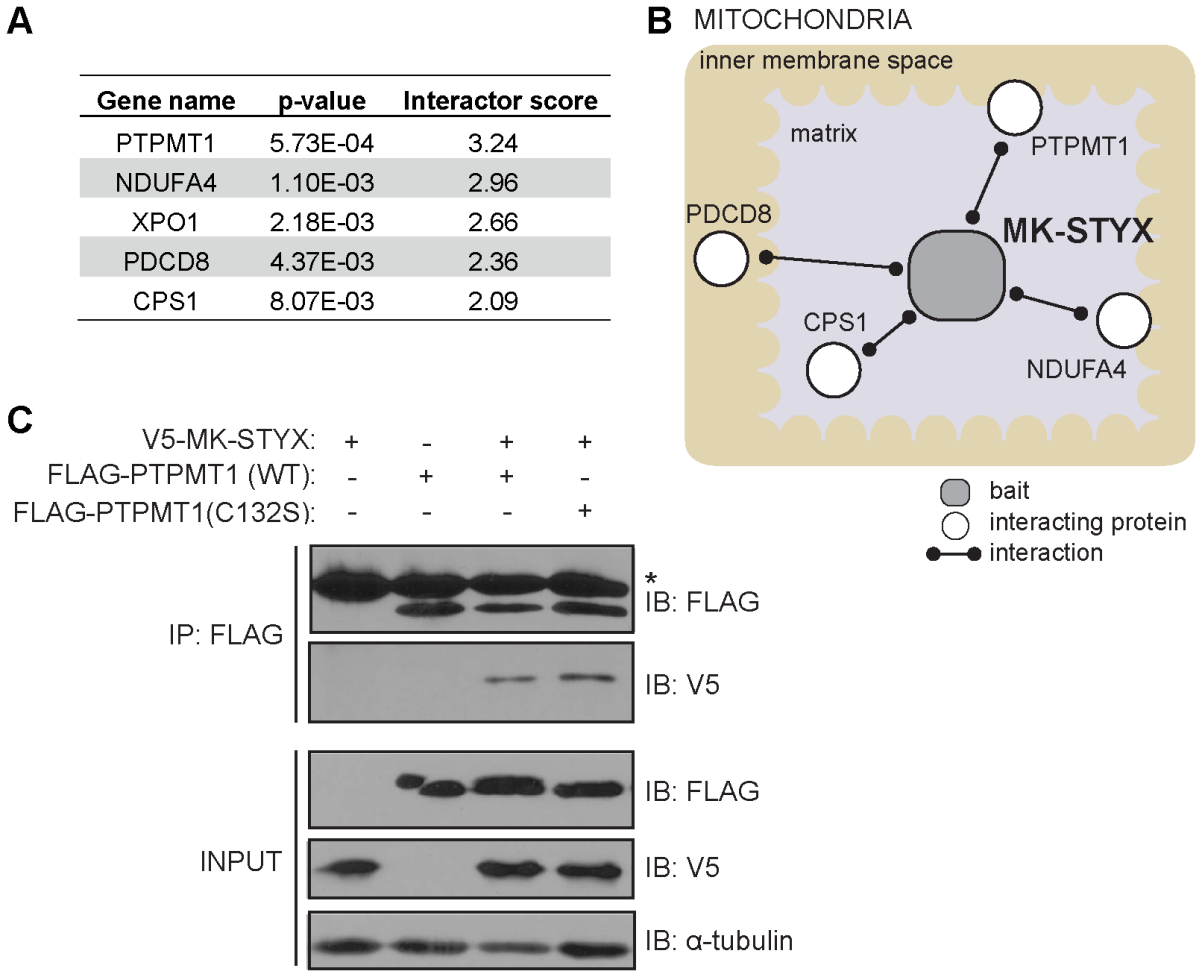
MK-STYX interacts with PTPMT1. (A) TAP-tagged MK-STYX was expressed and immunopurified from HeLa cells. Endogenous interaction partners were identified by LC-MS/MS and were bioinformatically filtered against a large control dataset (∼350 non-MK-STYX TAP tag experiments) to identify unique and significant interactions with MK-STYX. The gene name of each protein identified is listed in order of significance (the most significant hits listed at the top). A p value (demonstrating the significance of each interaction partner), and interactor score (calculated based on the p value, number of replicates in which the interactor was identified, uniqueness of the MK-STYX interaction relative to the control interactions, and MASCOT scores and total coverage of the peptides) is shown for each interactor (lower p-value and/or higher interactor score correlates with a stronger molecular interaction with MK-STYX). (**B**) The mitochondrial localization of top MK-STYX interaction partners is shown (gray rectangle: bait; white circle: interaction partner; line: interaction). (**C**) V5-MK-STYX and FLAG-PTPMT1 (wildtype) or FLAG-PTPMT1 C132S (a catalytically inactive mutant) were transfected into 293FT cells and FLAG immunoprecipitated. Input lysates or immunoprecipitates were immunoblotted with the indicated antibodies. The * indicates light chain of the antibody used to immunoprecipitate the protein.

To generate confidence in our data sets, we performed each TAP experiment (N- and C-tagged) in triplicate for a total of six independent experimental conditions. Upon expression of these constructs in HeLa cells, MK-STYX was isolated by a two-step affinity purification method, interacting proteins were resolved by SDS-PAGE, and gel fragments were digested and identified by liquid chromatography coupled to mass spectrometry (LC-MS/MS). Unique interactors were identified as those peptides corresponding to proteins identified in at least one of three replicates across the experimental conditions. To determine specific interactors (relative to non-specific binding proteins), all co-interacting peptides identified were bioinformatically filtered against a database of approximately 350 control (non-MK-STYX) TAP-tagging experiments. Statistical analysis eliminated these non-specific interacting proteins and prioritized the most unique interacting proteins with MK-STYX (**Table S1**). MS data was also used to compile an ‘interactor score’, which evaluated the number of experiments in which the peptide was found (out of six total); the number of peptides correlating with a protein (to increase confidence in the interactor); and MASCOT scores (Matrix Science) of those peptides (demonstrating the reliability of the protein identification). Thus, a larger interactor score correlates with a more robust proteomic interaction. The protein with the highest interactor score in our experiment was MK-STYX, which was expected as TAP experiments commonly show the most peptide coverage from the baited protein. The most significant (highest interactor score) and unique (p<0.0006) interaction partner of MK-STYX was the mitochondrial dual specificity phosphatase, PTPMT1 (**Figure 1A**). In addition, several proteins that localize to the mitochondrial matrix or IMM were identified as significant interactors, consistent with our previously report characterizing MK-STYX localization to the mitoplast (**Figure 1B**) [4]. Due to the overlapping mitochondrial localization of MK-STYX and PTPMT1, and the precedence for catalytically inactive phosphatases regulating active phosphatases, we further validated this interaction on a genetic, physical, and functional level.

### Characterization of the MK-STYX – PTPMT1 physical interaction

Our initial TAP-tagging experiment suggested that MK-STYX and PTPMT1 physically interact. We subsequently created epitope-tagged overexpression constructs of both genes to confirm that these proteins could interact in cells. We expressed V5-tagged MK-STYX, FLAG-tagged PTPMT1, or simultaneously co-expressed these constructs and performed co-immunoprecipitations. Immunoprecipitation of FLAG-tagged PTPMT1 from cells co-expressing both PTPMT1 and MK-STYX demonstrated an interaction with MK-STYX, as revealed by a band at 37 kDa, the molecular weight of V5-tagged MK-STYX (**Figure 1C**).

To further characterize the physical interaction between MK-STYX and PTPMT1, we generated a mutant form of the PTPMT1 overexpression construct that has been previously shown to disrupt enzymatic function [15]. We utilized this catalytically inactive mutant of PTPMT1 (C132S) to determine if the MK-STYX-PTPMT1 interaction was dependent upon the catalytic activity of PTPMT1. As shown in **Figure 1C**, catalytically inactive PTPMT1 interacted with MK-STYX with similar stoichiometry as the wild type PTPMT1-MK-STYX interaction, demonstrating that PTPMT1 does not require enzymatic activity for the two proteins to interact.

### MK-STYX reduces PTPMT1 phosphatase activity *in vitro*

**Figure 2:**
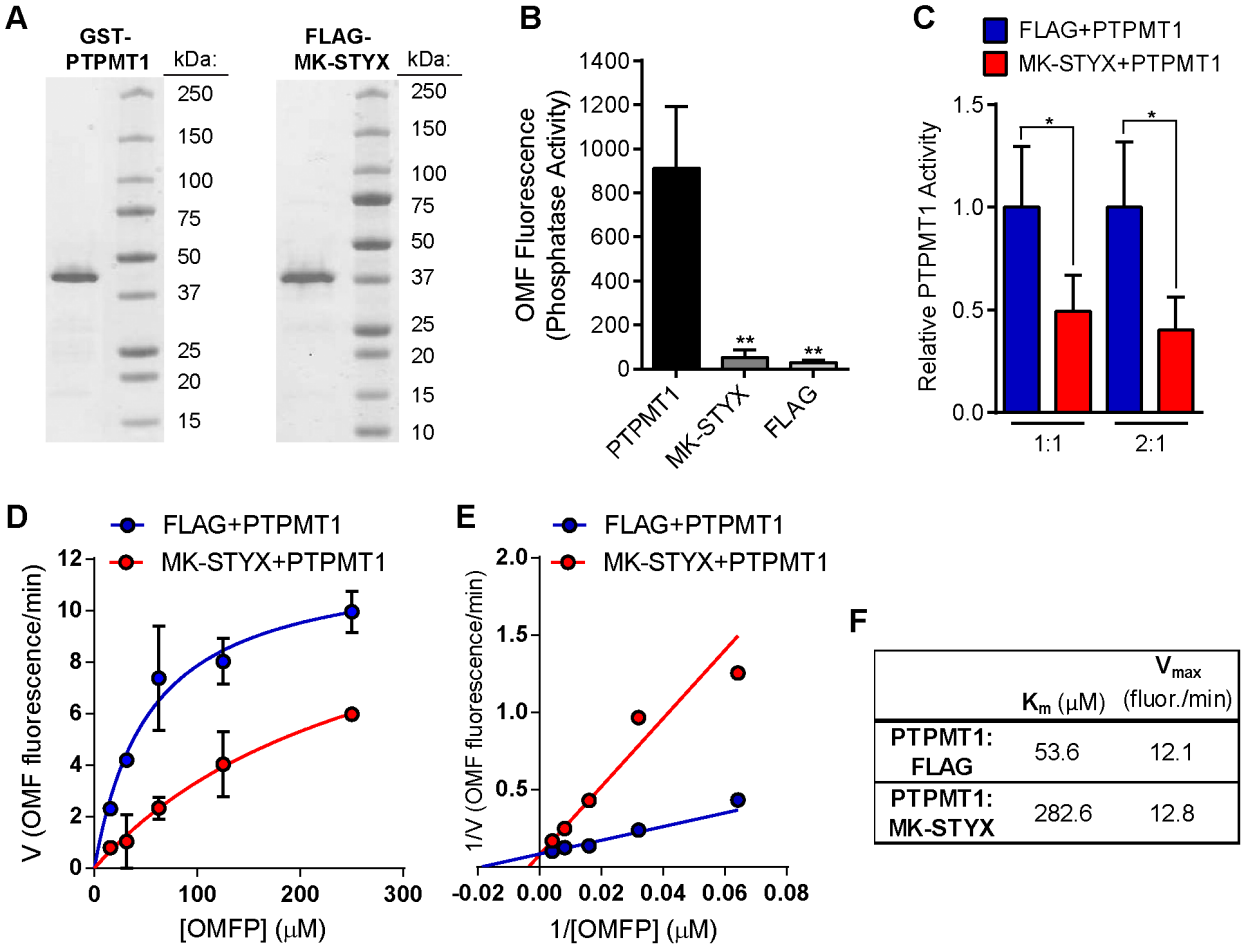
MK-STYX reduces PTPMT1 phosphatase activity *in vitro*. (**A**) GST-PTPMT1 (2 μg; left) or FLAG-MK-STYX (2 μg; right) was resolved by SDS-PAGE and coomassie stained alongside the indicated protein standards. (**B**) 2 μg GST-MKSTYX, FLAG-MKSTYX, or FLAG protein alone was incubated with OMFP at each of 4 concentrations (15.6, 31.3, 63.5, 125 μM) for 1 hour. Mean background-corrected OMF fluorescence was determined from all reactions. Bars represent standard deviation. Unpaired, two-tailed t-test for MK-STYX and FLAG each compared to PTPMT1: **p≤0.01. (**C**) FLAG protein (blue bars) or FLAG-MK-STYX (red bars) was incubated with GST-PTPMT1 at 1∶1 or 2∶1 mass ratios and relative PTPMT1 phosphatase activity determined using the four OMFP concentrations above and 40 minute reactions. Mean values are plotted; bars represent standard deviation. Unpaired, two-tailed t-tests: *p≤0.05. (**D**) PTPMT1 activity was measured kinetically in the presence of FLAG protein (blue) or FLAG-MK-STYX (red) using a series of OMFP concentrations and reactions at 20 to 40 minute intervals for 2 hours. Data points represent mean PTPMT1 enzymatic velocity (in OMF fluorescence per minute) from both 1∶1 and 2∶1 mass ratio reactions (as in C) derived by linear regression analysis. Bars represent standard deviation. Lines represent best-fit using Michaelis-Menten modeling. (**E**) A Lineweaver-Burk plot was generated by double reciprocal transformation of the data in (D). (**F**) K_m_ (in μM) and Vmax (in OMF fluorescence per minute) for PTPMT1 under each condition were determined from Michaelis-Menten plots.

Next, we sought to determine if MK-STYX alters the catalytic activity of PTPMT1. To this end, we generated pure recombinant GST-tagged PTPMT1 and FLAG-tagged MK-STYX proteins (**Figure 2A**). To enable a kinetic characterization of PTPMT1 activity, we chose to utilize 3-O-methylfluorescein phosphate (OMFP), a substrate that fluoresces upon dephosphorylation into OMF [16]. In addition to being amenable to a robust, kinetic characterization of PTPMT1, the K_m_ for OMFP was reported to be similar to that of a phospholipid substrate, phosphatidylinosital-5-phosphate (PI(5)P) [17]. We first confirmed that, in contrast to PTPMT1, MK-STYX does not harbor catalytic activity towards OMFP, yielding negligible OMF fluorescence similar to a negative control FLAG-only protein (**Figure 2B**). Next, we co-incubated PTPMT1 with either MK-STYX or FLAG protein, and determined phosphatase activity. Compared to FLAG protein alone, MK-STYX reduced PTPMT1 activity by 51% (p = 0.03) when incubated at a 1∶1 mass ratio, and 60% (p = 0.02) when incubated 2∶1 (**Figure 2C**).

To further characterize the nature of MK-STYX mediated PTPMT1 inhibition, we performed Michaelis-Menten kinetics [18], [19]. PTPMT1 activity was measured kinetically over the course of two hours using a series of OMFP concentrations (from 0 to 250 μM) in the presence of MK-STYX or a FLAG control. PTPMT1 activity (in OMF fluorescence generated per minute) at each OMFP concentration was derived by linear regression and best-fit Michaelis-Menten curves generated from the data (**Figure 2D**). In addition, reciprocal transformation of this data was used to generate a Lineweaver-Burk (or double-reciprocal) plot, which is useful for distinguishing modes of inhibition (**Figure 2E**) [20]. From these analyses, V_max_ (maximum enzyme velocity) and K_m_ (substrate concentration at 1/2 V_max_) were determined for each condition (**Figure 2F**).

We found that in the presence of a FLAG protein control, PTPMT1 had a K_m_ of 53.6 μM towards OMFP. This is slightly higher than the K_m_ of 39 μM reported previously and may reflect a modest effect of FLAG incubation alone [17]. Co-incubation with MK-STYX increased the apparent K_m_ over 5-fold (to 282.6 μM OMFP), while leaving V_max_ largely unchanged The significant increase in K_m_ caused by MK-STYX, evidenced by the steep trajectory in the Lineweaver-Burk plot (red line) when compared to the FLAG control (blue), is consistent with competitive inhibition. Through this mode of inhibition, MK-STYX is predicted to disrupt the interaction of OMFP with PTPMT1 [19], [20].

### MK-STYX knockdown does not protect cells from the viability loss caused by PTPMT1 depletion

We have previously characterized MK-STYX as a ‘death phosphatase’, demonstrating that its overexpression promotes apoptosis while its depletion causes striking resistance to cell death [4]. Conversely, we have classified PTPMT1 as a ‘survival phosphatase’ as its knockdown induces cell death in a large number of cancer cell lines [21]. These observations led us to hypothesize that MK-STYX and PTPMT1 functionally oppose one another in the regulation of cell death. Specifically, given the precedence of inactive phosphatases regulating active phosphatase binding partners and the fact that MK-STYX dampens PTPMT1 enzymatic activity *in vitro*, we hypothesized that MK-STYX functions upstream of PTPMT1, downregulating its activity to promote apoptosis.

**Figure 3:**
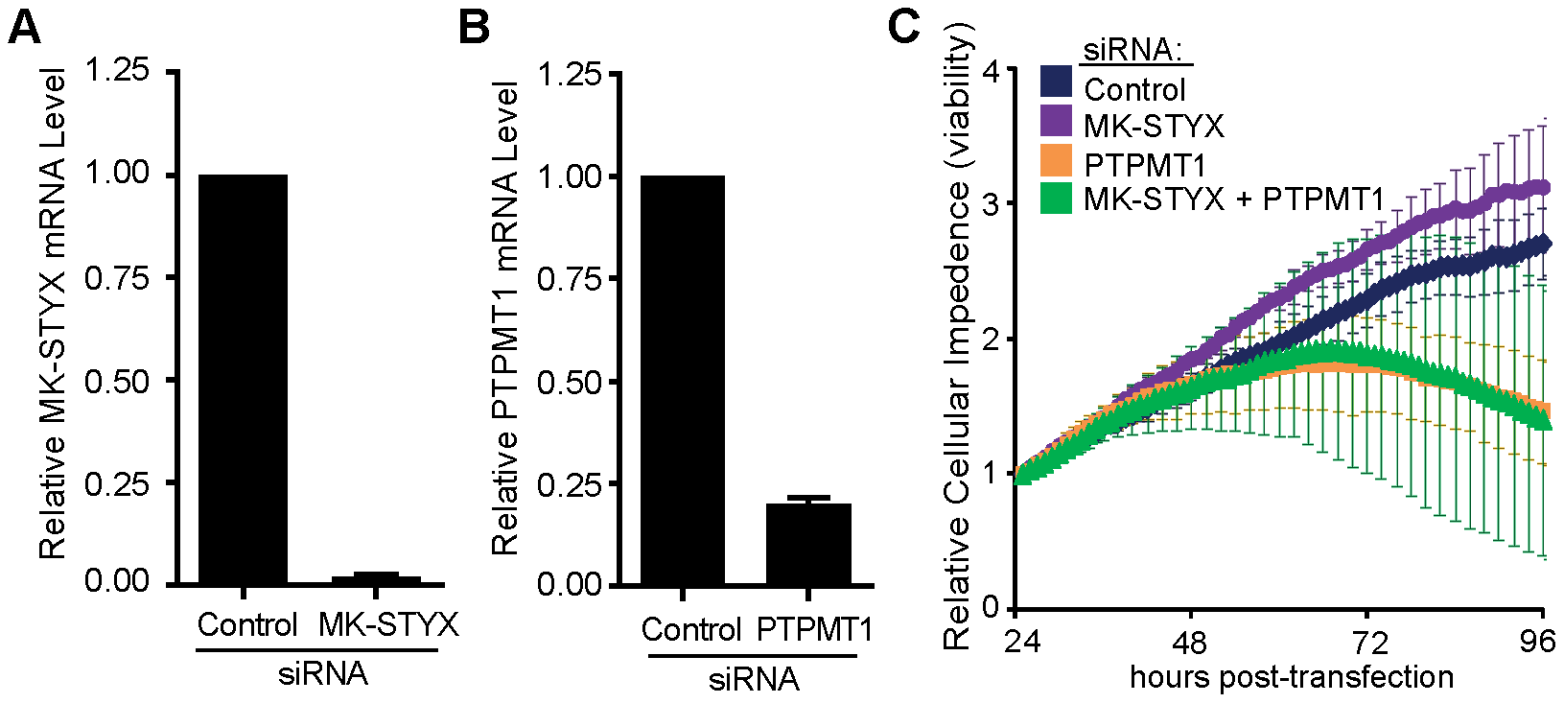
MK-STYX knockdown fails to protect cells from the loss of viability caused by PTPMT1 depletion. (**A–B**) HeLa cells were transfected with a pool of two unique MK-STYX siRNAs (A), a pool of two unique PTPMT1 siRNAs (B), or a non-targeting control siRNA (A and B) for 30 hours. Relative knockdown was assessed via RT-PCR. Values represent mean mRNA levels normalized to control siRNA-treated cells using HPRT1 as a reference gene. Error bars represent standard deviation. (**C**) HeLa cells were transfected with control, MK-STYX, PTPMT1, or MK-STYX + PTPMT1 siRNAs for 24 hours before cellular viability was assayed in real-time using a xCELLigence plate reader.

From this hypothesis, we first predicted that dual knockdown of MK-STYX and PTPMT1 would result in decreased basal cell viability, as MK-STYX depletion would not protect against cell death caused by the downregulation of its cellular target, PTPMT1 [22]. To test this hypothesis, we transfected HeLa cells with siRNA sequences targeting MK-STYX, PTPMT1, or both enzymes simultaneously (**Figure 3A-B**). We then monitored cell viability in real time using a xCELLigence plate reader, which captures electrical impedance from cells seeded on an electrode-containing 96-well plate. As cells proliferate and adhere to the electrode plate, impedance increases; likewise, as cells detach from the plate (due to cell death), impedance decreases. Thus, cellular impedance is an indirect measure of cellular proliferation and viability, which can be measured every hour over a span of five or more days [23]. We observed that cells transfected with control or MK-STYX-specific siRNA grew with similar kinetics (**Figure 3C**: blue and purple lines, respectively). In contrast, cells transfected with PTPMT1 siRNA showed signs of decreasing impedance, indicating cellular detachment and death beginning at 72 hours post-transfection, similarly to our previously published data (**Figure 3C**: orange line) [21]. Consistent with our hypothesis, the dual knockdown of MK-STYX and PTPMT1 caused cells to detach with similar kinetics to PTPMT1 knockdown cells (**Figure 3C**: green line). This demonstrates that MK-STYX is not required for the PTPMT1 knockdown phenotype and is consistent with, although not conclusive of MK-STYX functioning as an upstream regulator of PTPMT1.

### Dual knockdown of PTPMT1 and MK-STYX restores chemosensitivity

**Figure 4:**
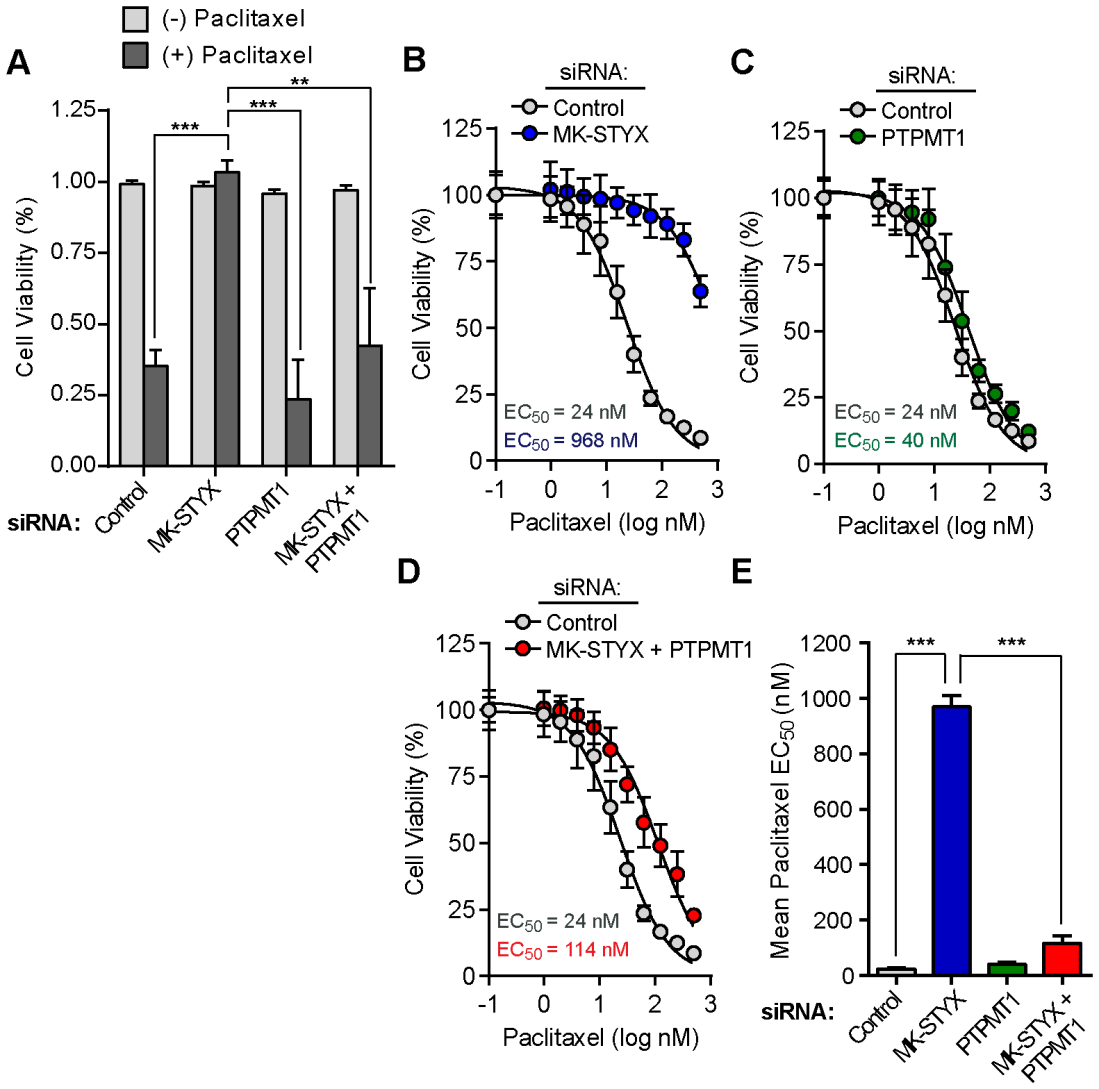
Dual knockdown of MK-STYX and PTPMT1 is sufficient to resensitize MK-STYX knockdown cells to chemotherapeutic treatment. (A) HeLa cells were plated on an electrode-containing plate and viability was measured using the xCELLigence system. Cells were transfected for 30 hours and incubated in the presence of 50 nM paclitaxel or a vehicle-only control for an additional 24 hours. Mean cell viability values following both drug (dark gray bars) and vehicle (light gray bars) treatments are shown. Bars represent standard deviation of at least triplicate measurements. Unpaired, two-tailed t-tests: **p≤0.01; ***p≤0.001. (B–E) HeLa cells were transfected with control (B–D, gray circles), MK-STYX (B, blue circles), PTPMT1 (C, green circles), or MK-STYX+PTPMT1 siRNAs (D, red circles) for 30 hours before being treated with a dose response of paclitaxel for an additional 24 hours. Mean EC_50_ values were determined for each condition using GraphPad PRISM (plotted in E; indicated on each plot). Bars represent standard deviation of four measurements per condition. Unpaired, two-tailed t-tests: ***p≤0.001.

Cells transfected with MK-STYX siRNA are significantly chemoresistant [2], [4]. The epistatic nature of PTPMT1 in our dual knockdown experiments measuring basal cell viability led us to next predict that PTPMT1 knockdown may be sufficient to resensitize MK-STYX-knockdown cells to chemotherapeutics. We used the same siRNA transfection approach, outlined above, to determine the effects of knockdown on chemotherapeutic sensitivity with the key exception that cells were treated with paclitaxel at 30 hours post-knockdown before PTPMT1 knockdown itself compromises viability. The cells were incubated in the presence of chemotherapeutic for an additional 24 hours; the entire siRNA knockdown spanned only 54 hours, a time point at which untreated PTPMT1 knockdown cells are still viable (**Figure 4A**: dark gray bars). As expected, 50 nM paclitaxel treatment resulted in cell death in cells transfected with a control siRNA for 24 hours (**Figure 4A**: light gray bars). Notably, MK-STYX knockdown promoted robust chemoresistance, as there was little difference between untreated and paclitaxel-treated MK-STYX knockdown cells. Cells transfected with PTPMT1 siRNA underwent cell death with similar kinetics to control cells. Dual knockdown cells (co-transfected with MK-STYX and PTPMT1 siRNAs) were also susceptible to paclitaxel treatment, with cell death approximately equivalent to control- and PTPMT1-siRNA transfected cells.

In order to fully understand the chemosensitivity profile of dual knockdown cells, we transfected HeLa cells with siRNAs and generated dose-response curves with paclitaxel. We utilized concentrations of paclitaxel ranging from 0.1 to 500 nM, as it broadly covers the viability profile of HeLa cells, which have an EC_50_ of 24 nM (**Figure 4B–D**: gray circles). MK-STYX knockdown cells demonstrated robust chemoresistance, with an EC_50_ value of 968 nM - over 40-fold higher than cells transfected with a control siRNA (**Figure 4B**: blue circles). PTPMT1 siRNA had minimal effect on paclitaxel efficacy, yielding an EC_50_ value of 40 nM (**Figure 4C**: green circles). PTPMT1 knockdown in the context of MK-STYX knockdown, however, also showed a low EC_50_ value for paclitaxel (114 nM), particularly when compared to the EC_50_ value of MK-STYX knockdown cells (**Figure 4D**: red circles). Analysis of the results of four independent dose-response curves per siRNA treatment revealed a statistically significant difference in the EC_50_ value of cells transfected with both MK-STYX and PTPMT1 siRNAs compared to those transfected with MK-STYX siRNA alone (**Figure 4E**). These data demonstrated that PTPMT1 expression is critical for the chemoresistant phenotype seen in MK-STYX knockdown cells, and suggested that PTPMT1 is epistatic to MK-STYX, functioning downstream in the same molecular signaling pathway that modulates cellular viability.

### Dual knockdown of PTPMT1 and MK-STYX restores cytochrome c release

**Figure 5:**
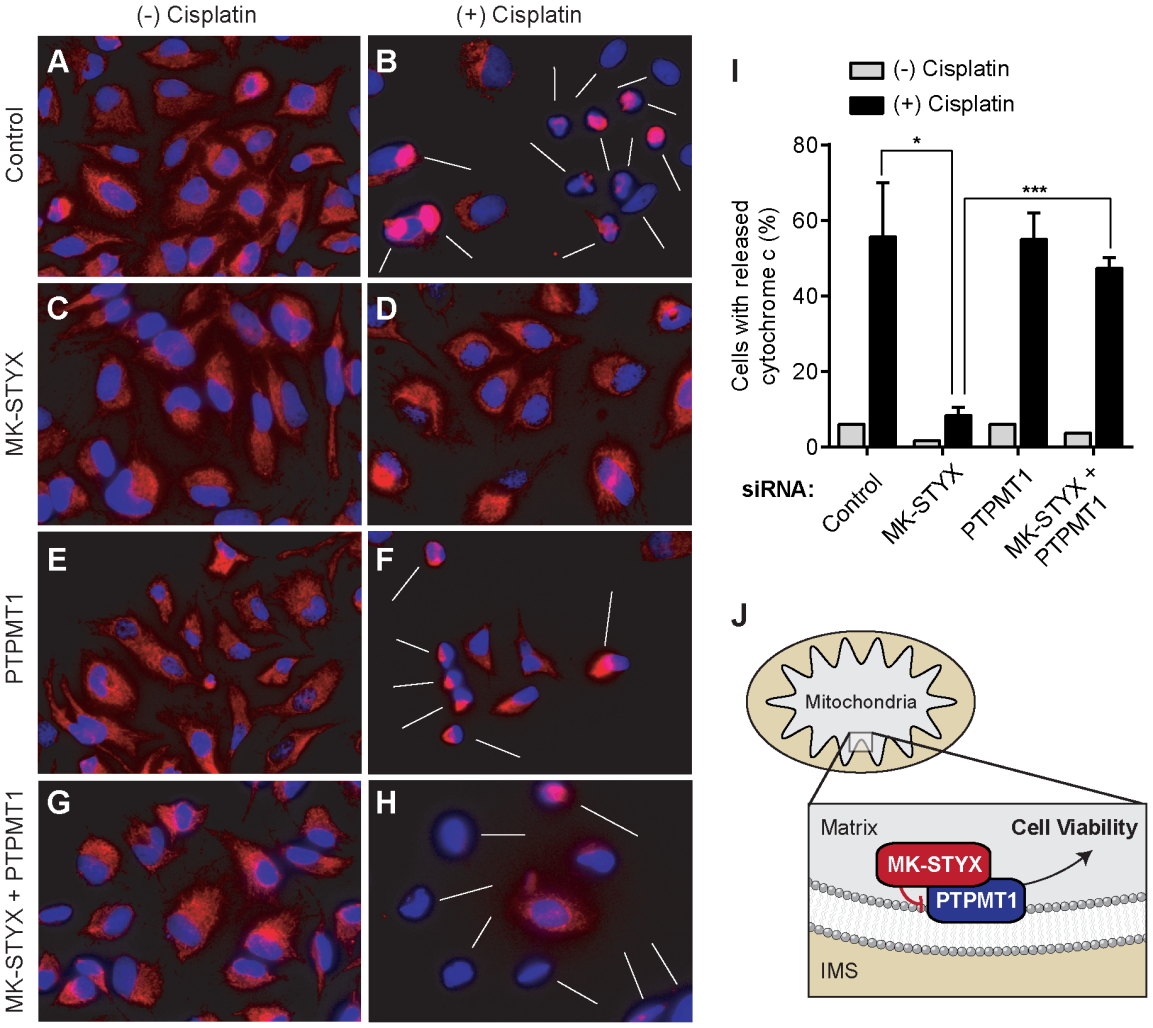
Dual knockdown of MK-STYX and PTPMT1 resensitizes MK-STYX knockdown cells to cytochrome c release. (**A–H**) HeLa cells were transfected with control, MK-STYX, PTPMT1, or MK-STYX+PTPMT1 siRNAs for 30 hours before being treated with 50 μM cisplatin or a vehicle only control for an additional 24 hours. All cells were incubated with the pan-caspase inhibitor zVAD (20 μM) while being subjected to drug treatment to allow visualization of cells post-drug treatment. After 24 hours, cells were washed, fixed, permeabilized, and stained. Cytochrome c localization was identified in cells via indirect immunofluorescence (red); punctate perinuclear staining indicates healthy cells with intact mitochondria; a loss of signal or very small, bright punctae near the nucleus reflects cells that have undergone cytochrome c release (indicated by white arrows in (B), (F), and (H)). Nuclei were labeled using Hoechst stain to allow visualization of cells with released cytochrome c. (**I**) Populations of cells undergoing cytochrome c in each siRNA condition (untreated: gray bars; cisplatin-treated: black bars) were quantified by blindly assigning a score of 0 (unreleased cytochrome c) or 1 (released cytochrome c) for at least 100 cells/field. Bars represent standard deviation of 3 measurements. Unpaired, two-tailed t-tests: ***p≤0.05; ***p≤0.001. (**J**) Our working model suggests that MK-STYX physically binds and suppresses the catalytic activity of PTPMT1, a matrix-facing IMM protein that promotes cell viability.

We have previously shown that MK-STYX knockdown promotes chemoresistance by blocking intrinsic apoptosis, specifically at the level of cytochrome c release [4]. Our data demonstrating that the PTPMT1 downregulation in MK-STYX knockdown cells resensitizes these cells to chemotherapeutic treatment suggests that these cells may also be resensitized to cytochrome c release. To test this hypothesis, we transfected cells with control, MK-STYX, PTPMT1, or MK-STYX plus PTPMT1 siRNAs for 30 hours before treating them with 50 μM cisplatin, or a vehicle control for an additional 24 hours. As shown in **Figures 5A, C, E, and G**, transfection of any of these siRNAs failed to significantly compromise cellular viability; fewer than 10% of cells in any of the four conditions released cytochrome c (**Figure 5I**). This is particularly notable in the cells transfected with PTPMT1 siRNA, as a time point was selected to assay PTPMT1-mediated resensitization before PTPMT1 knockdown itself causes cellular toxicity (**Figures 5E and I**).

After treatment with 50 μM cisplatin for 24 hours, robust cell death was shown in cells transfected with a non-targeting control (**Figure 5B**). Consistent with our previous data, MK-STYX knockdown significantly reduced the number of cells with released cytochrome c in these conditions, reflecting the stark chemoresistant phenotype of these cells (p<0.01, **Figures 5D and I**). PTPMT1 knockdown did not significantly alter cisplatin-induced cytochrome c release relative to cells transfected with a control siRNA, which was consistent with our data demonstrating comparable paclitaxel dose response curves (**Figure 5F**). The dual knockdown of MK-STYX and PTPMT1 resulted in a population of cells undergoing cytochrome c release comparable to cells transfected with either a control or PTPMT1 siRNA alone, with statistically insignificant differences in cytochrome c release (**Figures 5H, I**). Increased cytochrome c release in the dual knockdown cells is significant relative to cells transfected with MK-STYX siRNAs alone (p<0.001), demonstrating that PTPMT1 expression is required to block cytochrome c release in MK-STYX knockdown cells (**Figure 5I**). These data, along with the data represented in Figures 2 and 3 suggested that PTPMT1 is epistatic to MK-STYX, and its function is required for the sustained viability of cells with downregulated MK-STYX.

## Discussion

Here, we have shown that the catalytically inactive mitochondrial phosphatase, MK-STYX, interacts with PTPMT1 on a physical, genetic, and functional level. We demonstrated that PTPMT1 expression is required for chemoresistance in cells depleted of MK-STYX, and that PTPMT1 knockdown is sufficient to restore an apoptotic response in MK-STYX knockdown cells.

We have confirmed a physical interaction between MK-STYX and PTPMT1 in cancer cells. MK-STYX lacks catalytic activity due to a naturally occurring cysteine-to-serine substitution, rendering the enzyme incapable of dephosphorylation [3]. We confirmed this lack of phosphatase activity using recombinant MK-STYX and OMFP substrate. Interestingly, this mutation does not impair substrate binding and, in fact, is used as an experimental tool to generate “substrate-trapping mutants” of active phosphatases [24]. This suggests that MK-STYX may function as an endogenous substrate-trapping enzyme. In support of this, binding of MK-STYX to its only previously reported interaction partner, G3BP1 (GTPase Activating Protein (SH3 domain) Binding Protein 1), was abrogated when MK-STYX was mutated back to an active enzyme [25]. This raises the possibility that MK-STYX may bind a phosphorylated residue on PTPMT1 to mediate this interaction. Interestingly, PTPMT1 was recently identified as phosphorylated at Ser35, a residue which may facilitate the MK-STYX interaction [26]. Experiments to fully characterize this interaction will provide important mechanistic insight into the function of these enzymes.

In addition to physically interacting in cells, we discovered that MK-STYX significantly reduces the phosphatase activity of PTPMT1. This data was obtained using purified recombinant proteins *in vitro*, supporting the conclusion that this inhibitory relationship is direct. Specifically, the data best supports a competitive mode of PTPMT1 inhibition conferred by MK-STYX [20]. The substantial increase in K_m_ caused by co-incubation with MK-STYX suggests MK-STYX can interfere with substrate binding to free PTPMT1. While competitive inhibitors (especially small molecules) often bind an enzyme in the same location as the substrate (i.e., the active site) and thereby directly compete with it, this is not always the case [20]. It is possible for competitive inhibition to be achieved through distant binding and conformational regulation [20]. Accordingly, these data are consistent with MK-STYX binding PTPMT1 at a location distal to the active site, such as at Ser35, and impeding the ability of PTPMT1 to interact with phosphorylated substrates. That said, it is important to emphasize that enzymatic data were collected using recombinant proteins (with unknown post-translational modifications) and a generic substrate. While this analysis provides strong evidence for the directness of the interaction, the relevancy of the mechanism of inhibition described here *in vivo* remains to be determined. Further studies, including structural modeling and assays that more closely reflect the endogenous activity of PTPMT1, are required to further clarify the nature of this relationship.

Consistent with the direct repression of PTPMT1 activity by MK-STYX, we have demonstrated that MK-STYX and PTPMT1 dually function to regulate apoptosis and chemosensitivity, with PTPMT1 functioning downstream of MK-STYX (**Figure 5J**). PTPMT1 was recently discovered to dephosphorylate phosphatidylglycerol phosphate (PGP) in the penultimate step of mammalian cardiolipin synthesis [27]. Accordingly, PTPMT1 depletion leads to PGP accumulation and reduced cardiolipin content in cells [21], [27]. Consistent with our data demonstrating that PTPMT1 knockdown increases cell death and chemosensitivity, several reports have shown that reduced cardiolipin renders cells sensitive to apoptotic stimuli [28], [29]. When cardiolipin is depleted, cytochrome c is liberated into the intermembrane space (IMS), priming its release in the “point of no return” stage of apoptosis. Furthermore, the absence of cardiolipin compromises mitochondria cristae morphology and reduces efficiency of the electron transport chain [30]. Together, these mitochondrial defects may increase vulnerability to apoptosis in the absence of phospholipids, such as cardiolipin.

Important future studies include determining whether MK-STYX depletion perturbs the levels of mitochondrial phospholipids, including cardiolipin, and establishing whether these changes are required for the chemoresistant phenotype of MK-STYX knockdown cells. If so, this may represent a novel pathway which cancer cells could exploit to resist chemotherapeutic treatment. Accordingly, targeting mitochondrial lipid metabolism may represent a potential therapeutic strategy for some chemoresistant tumors. Future challenges will include identification of increased cardiolipin or other phospholipids as a mechanism of chemoresistance *in vivo*, in both animal models and patient derived tumor samples.

## Materials and Methods

### Cells and Reagents

HeLa cells (ATCC) and 293FT cells (Invitrogen) were cultured in DMEM with a final concentration of 10% FBS. Paclitaxel (Sigma-Aldrich), as well as cisplatin and the pan-caspase inhibitor zVAD (Calbiochem) were resuspended in DMSO according to manufacturer directions. 3-O-Methylfluorescein phosphate (OMFP) was purchased from Sigma-Aldrich, diluted initially in DMSO to 10 mM, then to final concentration in phosphatase assay buffer. Recombinant proteins were obtained from BPS Bioscience (see below). FLAG peptide was obtained from Sigma-Aldrich.

### siRNA Transfection

Control (AllStars Negative Control), MK-STYX (siRNA #1: SI02224691; siRNA #2: SI02659167), or PTPMT1 (siRNA #1: SI00687183; siRNA #2: SI00687204) (Qiagen) were transfected into HeLa cells at a concentration of 50 nM per siRNA with Oligofectamine (Invitrogen) for the time indicated in each figure legend. Knockdown efficiency was assayed using RT-PCR and the delta-delta Ct method using HRPT1 as a reference gene, as previously described [2].

### Recombinant Protein Purification

GST-PTPMT1 and FLAG-MK-STYX (Styxl1) were obtained from BPS Bioscience (San Diego, CA). Briefly, PTPMT1 (NM_175732; aa 2-201 (end)) fused to an amino-terminal GST tag was produced using a baculovirus infected Sf9 insect cell system. High yield (0.6 mg per ml) and purity (≥85%) was obtained. Similarly, MK-STYX (STYXL1; NM_016086; aa 2-313 (end)) fused to an amino-terminal FLAG tag was produced using a baculovirus infected Sf9 insect cell system. High yield (0.15 mg per ml) and purity (≥90%) was obtained.

### Phosphatase Assays

A 2× stock of phosphatase assay buffer, pH 7.0 was prepared in water (0.2 M sodium acetate, 0.1 M bis-tris, 0.1 M tris base) and 2× (10 mM) dithiothreitol (DTT) added fresh before use (modified from [17]). Individual phosphatase reactions were prepared by combining 50 μl 2× phosphatase assay buffer, recombinant phosphatase (GST-PTPMT1 or FLAG-MK-STYX), OMFP, and water to 100 μl final volume. Reactions were incubated at 37°C for the times indicated in figure legends then transferred to a black-wall, clear bottom 96-well plate. Fluorescence was read immediately on a Synergy HT Multi-Mode Microplate Reader (Bio-Tek) using 485/20 nm excitation and 590/35 nm emission (bottom read). Background was determined as fluorescence from reactions without GST-PTPMT1 (i.e., substrate-only signal). 2 μg of GST-PTPMT1 was used in all reactions as it catalyzed linear product formation over the course of 1 to 2 hours, with sufficient signal:background ratios (data not shown). For kinetic studies, OMFP was used at a final concentration of 0, 15.6, 31.3, 62.5, 125, or 250 μM (see figure legends). Background-corrected values were imported into GraphPad PRISM, converted to relative activity (to PTPMT1; Figure 2B–C), or fit to Michaelis-Menten and Lineweaver-Burk Models (Figure 2D-E), as indicated in the text and figure legends.

### TAP-MS/MS and proteomic analysis

TAP-epitope-tagged MK-STYX was expressed in HeLa cells by retrovirus-mediated gene transfer. N- (NTAP) and C- (CTAP) terminal fusions were constructed, and viral stocks were generated in HEK293 Gag-Pol packaging cell lines. Expression levels of MK-STYX were gauged close to endogenous levels by adjusting the multiplicity of infection. MK-STYX was immunopurified in complex with its endogenous associated proteins using a modified TAP protocol [31]. Protein samples were separated by SDS-PAGE, in-gel trypsin digested as described [32]. All co-purifying proteins, specific (interactors) and non-specific (false positives) proteins were identified by LC-MS/MS. For identification of high confidence interactors, specificity and reproducibility of the raw MK-STYX interactor data was compared against a control data set (>344) of purifications performed in control datasets from HeLa or 293T cells, as previously described [14].

### Cellular viability assays

CellTiter-Glo (Promega) was used to detect cellular viability according to the manufacturer's directions. Real-time cell proliferation and viability was determined using the xCELLigence system, as previously described [4]. EC_50_ values were calculated with GraphPad PRISM using a log inhibitor vs. response (3-parameter) algorithm and a bottom constraint of 0% viability.

### Immunofluorescence and microscopy

Immunofluorescence for cytochrome c release was performed as previously described [4]. Briefly, cells were transfected with siRNAs for 30 hours before being treated with 50 μM cisplatin or DMSO (as a vehicle only control). Cells were simultaneously treated with 20 μM zVAD, a pan-caspase inhibitor that allows visualization of cells after cytochrome c release. Cells were fixed in 4% paraformaldehyde, permeabilized in 0.2% Triton-X 100, and blocked in 5% goat serum for 1 hour. Cells were then incubated with an AF555-conjugated cytochrome c antibody (dilution 1∶100) (BD Biosciences) for 1 hour at room temperature (protected from light). Nuclei were counterstained with Hoechst stain. Imaging was performed on an epifluorescent microscope (Nikon Eclipse Ti), and images were captured and analyzed using the NIS Elements AR 3.10 software. To quantify cytochrome c release, plates were scored as punctate (0) or diffuse (1) for at least 100 different cells in random fields, and the mean percentage of diffusely-stained cells determined. Quantification is representative of three independent experiments.

### Statistical Analyses

p-values were generated by unpaired, two-tailed t-tests in which equal variance was not assumed (using Microsoft Excel or GraphPad PRISM). Brackets on figures indicate which two values were compared in each test and astericks indicate the following p-values: *  = p≤0.05; **  = p≤0.01; ***  = p≤0.001.

### Expression Constructs

The open reading frame (ORF) of human MK-STYX (STYXL1; NM_160862.2) was cloned into pRK7 with an N-terminal V5 tag (introduced in the forward primer). The ORF of human PTPMT1 (NM_175732.2) was cloned into pRK7 with a C-terminal FLAG tag (introduced in the forward primer). Cysteine 132 of PTPMT1 was mutated Serine (C132S) using a QuikChange Site-Directed Mutagenesis kit (Agilent) and sequence-verified.

### Immunoprecipitation

V5-MK-STYX and FLAG-PTPMT1 constructs were transfected into 293FT cells using FuGENE 6 according to the manufacturer's directions. 48 hours post-transfection, cells were lysed and cleared as previously described [4]. Proteins were immunoprecipitated after incubation of each lysate with 2 μg anti-FLAG antibody for 2 hours at 4°C with constant rotation. Immunopurified proteins were then captured by adding 40 μl of a 50% slurry of pre-blocked Protein G beads, which were incubated for 1 hour at 4 °C with constant rotation. Beads were gently collected via centrifugation and washed three times, twice with 1% NP40 in PBS and once with TNE buffer. Beads were then resuspended in 30 μL 2× Laemmli sample buffer, boiled, run on an SDS-PAGE gel before being transferred onto nitrocellulose for subsequent immunoblotting.

## Supporting Information

Table S1 **MK-STYX interactors identified by LC-MS/MS.** Endogenous interaction partners were identified by LC-MS/MS and bioinformatic filters applied against a large control dataset (∼350 non-MK-STYX TAP tag experiments) to identify unique and significant interactions with MK-STYX (STYXL1 gene symbol). The gene name of each protein identified is listed in order of significance (the most significant hits listed at the top and decreasing in significance down the list). Control hits designates how many peptides of that protein were found in any other non-MK-STYX TAP experiment, which is converted to a fraction of total TAP experiments in the next column. STYXL1 hits shows the amount of peptides from the identified protein were found in the TAP-MK-STYX experiment, which is also converted to a fraction relative to number of replicates in the experiment. Number of IDs shows in how many experiments (of the triplicate performed) the peptides corresponding to the interacting protein was found. These data were compiled to create a p-value, demonstrating the significance of each interaction partner. Additionally, an interactor score was calculated based on this p-value, the number of replicates in which the interactor was identified, the uniqueness of the MK-STYX interaction relative to the control interactions, as well as the MASCOT scores and total coverage of the peptides identified from each interaction protein. A lower p-value and/or a higher interactor score correlates with a stronger molecular interaction with MK-STYX.
(DOCX)
