# Supplementary material for: The Pseudophosphatase MK-STYX Physically and Genetically Interacts with the Mitochondrial Phosphatase PTPMT1
Source: PLoS One. 2014 Apr 7;9(4):e93896. doi: 10.1371/journal.pone.0093896 (PMC3977970; doi:10.1371/journal.pone.0093896)
Supplement: Table S1 — MK-STYX interactors identified by LC-MS/MS. Endogenous interaction partners were identified by LC-MS/MS and bioinformatic filters applied against a large control dataset (∼350 non-MK-STYX TAP tag experiments) to identify unique and significant interactions with MK-STYX (STYXL1 gene symbol). The gene name of each protein identified is listed in order of significance (the most significant hits listed at the top and decreasing in significance down the list). Control hits designates how many peptides of that protein were found in any other non-MK-STYX TAP experiment, which is converted to a fraction of total TAP experiments in the next column. STYXL1 hits shows the amount of peptides from the identified protein were found in the TAP-MK-STYX experiment, which is also converted to a fraction relative to number of replicates in the experiment. Number of IDs shows in how many experiments (of the triplicate performed) the peptides corresponding to the interacting protein was found. These data were compiled to create a p-value, demonstrating the significance of each interaction partner. Additionally, an interactor score was calculated based on this p-value, the number of replicates in which the interactor was identified, the uniqueness of the MK-STYX interaction relative to the control interactions, as well as the MASCOT scores and total coverage of the peptides identified from each interaction protein. A lower p-value and/or a higher interactor score correlates with a stronger molecular interaction with MK-STYX. (DOCX) [file pone.0093896.s001.docx]

**Table S1.**

| **Gene Name** | **Control Hits** | **Control Fraction** | **STYXL1 Hits** | **STYXL1 Fraction** | **# IDs** | **p-value** | **Interactor Score** |
| --- | --- | --- | --- | --- | --- | --- | --- |
| STYXL1 | 3 | 0.009 | 0 | 0.000 | 3 | 2.82E-06 | 5.549 |
| PTPMT1 | 27 | 0.078 | 24 | 0.070 | 3 | 5.73E-04 | 3.242 |
| NDUFA4 | 34 | 0.098 | 31 | 0.090 | 3 | 1.10E-03 | 2.960 |
| XPO1 | 8 | 0.023 | 6 | 0.017 | 2 | 2.18E-03 | 2.662 |
| PDCD8 | 12 | 0.035 | 10 | 0.029 | 2 | 4.37E-03 | 2.360 |
| CPS1 | 68 | 0.196 | 65 | 0.189 | 3 | 8.07E-03 | 2.093 |
| TUBB3 | 69 | 0.199 | 66 | 0.192 | 3 | 8.42E-03 | 2.075 |
| ARF4 | 22 | 0.063 | 20 | 0.058 | 2 | 1.30E-02 | 1.887 |
| COPA | 22 | 0.063 | 20 | 0.058 | 2 | 1.30E-02 | 1.887 |
| CCT8 | 84 | 0.242 | 81 | 0.235 | 3 | 1.50E-02 | 1.825 |
| NDUFS3 | 93 | 0.268 | 90 | 0.262 | 3 | 2.02E-02 | 1.695 |
| NDUFS7 | 97 | 0.280 | 94 | 0.273 | 3 | 2.28E-02 | 1.642 |
